# The complete mitochondrial genome of *Tetraclita japonica* (Crustacea: Maxillopoda: Sessilia) from Zhejiang (China) and phylogeny within Sessilia

**DOI:** 10.1080/23802359.2020.1791756

**Published:** 2020-07-20

**Authors:** Sheng Mao, Tian Ge, Panpan Chen, Yuefeng Cai, Nanjing Ji, Xin Shen

**Affiliations:** a Jiangsu Institute of Marine Resources/Jiangsu Key Laboratory of Marine Biotechnology, Jiangsu Ocean University, Lianyungang, China; b Co-Innovation Center of Jiangsu Marine Bio-industry Technology, Jiangsu Ocean University, Lianyungang, China

**Keywords:** Sessilia, *Tetraclita japonica*, mitochondrial genome, selection pressure, phylogeny

## Abstract

The complete mitochondrial genome of *Tetraclita japonica* (Crustacea: Maxillopoda: Sessilia) from Zhejiang (China) was presented (*T. japonica* ZJ). The genome is a circular molecule of 15,192 bp, all non-coding regions are 694 bp in length, and the longest one (307 bp) is located between *12S rRNA* and *trnK*. Phylogenetic analysis based on mitochondrial PCGs shows that *T. japonica* ZJ clusters with *T. japonica* CN, and they grouped with *T. japonica* (BP = 100), then grouped with *Tesseropora rosea*, *Tetraclita serrata*, *Tetraclita rufotincta*, *Epopella plicata*, and *Tetraclitella divisa* successively . The phylogenetic tree indicated that the Balanidae and Archaeobalanidae are polyphyletic, while the Chthamalidae is monophyletic. The result can help us to understand the phylogenetic history within Sessilia.

Barnacles are widely distributed, which can be found from the intertidal zone to the subtidal zone in almost any sea area (Newman and Ross 1976; Tsang et al. 2012). They are numerous and generally live together. Mature barnacles are the only species fixed on life among Crustacean. Tetraclitidae is a common family among barnacles. In this study, the sample of *Tetraclita japonica* was collected from Zhoushan Islands (N: 29.88, E: 122.41), located at Zhejiang Province, China, which was deposited in Jiangsu Key Laboratory of Marine Biotechnology with 95% ethanol. Total DNA was extracted from the muscle tissue of the sample with the TIANamp DNA Kit (TIANGEN, Beijing, China), which was stored at the Marine Museum of Jiangsu Ocean University (Accession number: TjapZJ-005).

The mitochondrial genome of *T. japonica* ZJ is 15,192 bp (GenBank accession number: MN891699), a circular DNA molecule that contains 13 protein-coding genes, 22 transport RNA genes, and 2 ribosomal RNA genes. Nine PCGs and 15 tRNAs are located at the heavy strand and remaining genes are located at the light strand. Analysis result on the proportion of nucleotides shows that the contents of A, C, G, and T are 34.5%, 21.3%, 12.5%, and 31.7%, respectively. The contents of A + T among 13 PCGs range from 58.5% to 71.4%, and the contents of *12S rRNA* and *16S rRNA* are 64.7% and 71.9%, respectively. All non-coding regions are 694 bp in length, and the longest one (307 bp) located between *12S rRNA* and *trnK* speculated the control region. All PCGs in *T. japonica* ZJ start with “ATN” (N = A, T, C, or G). Particularly, *nad5* ended by TAG, *cox3*, *nad3*, and *nad4* ended by T– and the remaining PCGs ended by TAA as the complete stop codon. The values of *Ka*/*Ks* on *cox1*, *cox2*, *cob*, *nd4l*, *atp6*, and *atp8* are all 0. Remarkably, the values of *Ka*/*Ks* on remaining PCGs range from 0.019 to 0.145 (*nd4* < *nd1* < *nd2* < *nd5* < *nd6* < *cox3* < *nd3*). In comparison, Cytochrome oxidase subunit genes (except *cox3*) suffer stronger selection pressure than NADH dehydrogenase subunit genes, which is consistent with Sun’s research result (Sun, 2017).

For the purpose of clarifying the phylogenetic relationships between barnacles and the position of *T. japonica* ZJ within Tetraclitidae, a phylogenetic tree used by PhyloSuite was constructed based on amino acid sequences of 13 PCGs from complete mitochondrial genomes with 24 Cirripedia species (Shen, Chan, et al. 2015; Shen, Tsang, et al. 2015; Tsang et al. 2015; Wares 2015; Baek et al. 2016; Shen, Chan, et al. 2016; Shen, Chu, et al. 2016; Shen, Tsoi, et al. 2016; Shen et al. 2017). Within the Tetraclitidae family (Figure 1), *T. japonica* ZJ clusters with *T. japonica* CN into a branch (BP = 97), and they grouped with *T. japonica* with high support (BP = 100), then grouped with *Tesseropora rosea*, *Tetraclita serrata*, *Tetraclita rufotincta*, *Epopella plicata*, and *Tetraclitella divisa* successively with high support. Our phylogenetic tree indicated that the Balanidae and Archaeobalanidae are polyphyletic, while the Chthamalidae is monophyletic, which were consistent with the previous results (Song et al. 2017; Cai et al. 2018; Chen et al. 2019; Ge et al. 2019). The result can help us to the understanding the phylogenetic history within Sessilia.

**Figure 1.**
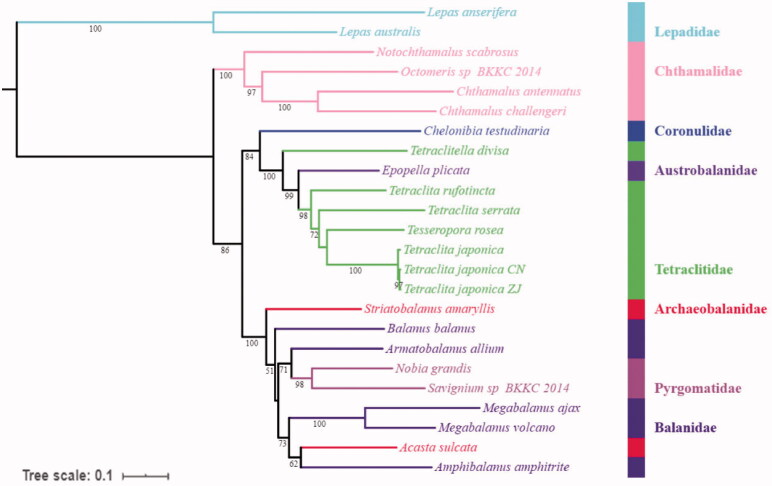
Phylogenetic tree based on 13 PCGs nucleotide acid sequences of *T. japonica* ZJ and 23 other Cirripedia mitochondrial genomes.

GenBank accession number used in this phylogenetic analysis: *Lepas anserifera*: NC_026576; *Lepas australis*: NC_025295; *Notochthamalus scabrosus*: NC_022716; *Octomeris* sp. BKKC-2014: KJ_754820; *Chthamalus antennatus*: NC_026730; *Chthamalus challenger*: NC_043920; *Chelonibia testudinaria*: NC_029169; *Tetraclitella divisa*: NC_029170; *Epopella plicata*:NC_033393; *Tetraclita rufotincta*: NC_037398; *Tetraclita serrata*: NC_029154; *Tesseropora rosea*: NC_037241; *Tetraclita japonica*: NC_008974; *Tetraclita japonica* CN: MH119183; *Tetraclita japonica* ZJ: MN891699; *Striatobalanus amaryllis*: NC_024526; *Balanus balanus*: NC_026466; *Armatobalanus allium*: NC_029167; *Nobia grandis*: NC_023945; *Savignium* sp. BKKC-2014: KJ_754821; *Megabalanus ajax*: NC_024636; *Megabalanus volcano*: NC_006293; *Acasta sulcate*: NC_029168; *Amphibalanus amphitrite*: NC_024525.

## Data Availability

The data that support the findings of this study are openly available in GenBank of NCBI at https://www.ncbi.nlm.nih.gov, reference number MN891699.
